# Assessment of diastolic function and atrial remodeling by MRI – validation and correlation with echocardiography and filling pressure

**DOI:** 10.14814/phy2.13828

**Published:** 2018-09-05

**Authors:** Felicia Seemann, Lauren A. Baldassarre, Fiorella Llanos‐Chea, Ricardo A. Gonzales, Karl Grunseich, Chenxi Hu, Lissa Sugeng, Judith Meadows, Einar Heiberg, Dana C. Peters

**Affiliations:** ^1^ Department of Radiology and Biomedical Imaging Yale University New Haven Connecticut; ^2^ Department of Clinical Physiology Skane University Hospital Lund University Lund Sweden; ^3^ Department of Biomedical Engineering Faculty of Engineering Lund University Lund Sweden; ^4^ Department of Cardiology Yale University New Haven Connecticut; ^5^ Department of Electrical Engineering Universidad de Ingenieria y Tecnologia ‐ UTEC Lima Peru; ^6^ San Francisco Department of Radiology and Biomedical Imaging University of California San Francisco California; ^7^ Section of Cardiovascular Medicine Department of Medicine Yale University New Haven Connecticut; ^8^ Wallenberg Center for Molecular Medicine Lund University Lund Sweden

**Keywords:** Cardiovascular magnetic resonance imaging, diastolic function, echocardiography, left atrial late gadolinium enhancement, left ventricular filling pressure

## Abstract

Atrial fibrosis can be estimated noninvasively by magnetic resonance imaging (MRI) using late gadolinium enhancement (LGE), but diastolic dysfunction is clinically assessed by transthoracic echocardiography (TTE), and rarely by MRI. This study aimed to evaluate well‐established diastolic parameters using MRI, and validate them with TTE and left ventricular (LV) filling pressures, and to study the relationship between left atrial (LA) remodeling and parameters of diastolic function. The study retrospectively included 105 patients (53 ± 16 years, 39 females) who underwent 3D LGE MRI between 2012 and 2016. Medical charts were reviewed for the echocardiographic diastolic parameters E, A, and e′ by TTE, and pressure catheterizations. E and A were measured from in‐plane phase‐contrast cardiac MRI images, and e′ by feature‐tracking, and validated with TTE. Interobserver and intraobserver variability was examined. Furthermore, LA volumes, function, and atrial LGE was correlated with diastolic parameters. Evaluation of e′ in MRI had strong agreement with TTE (*r* = 0.75, *P* < 0.0001), and low interobserver and intraobserver variability. E and A by TTE showed strong agreement to MRI (*r* = 0.77, *P* = 0.001; *r* = 0.73, *P* = 0.003, for E and A, respectively). Agreement between E/e′ by TTE and MRI was strong (*r* = 0.85, *P* = 0.0004), and E/e′ by TTE correlated moderately to invasive pressures (*r* = 0.59, *P* = 0.03). There was a strong relationship between LA LGE and pulmonary capillary wedge pressure (*r* = 0.81, *P* = 0.01). In conclusion, diastolic parameters can be measured with good reproducibility by cardiovascular MRI. LA LGE exhibited a strong relationship with pulmonary capillary wedge pressure, an indicator of diastolic function.

## Introduction

In a broad variety of cardiovascular diseases with poor outcomes, such as heart failure, aortic valve stenosis, hypertrophic cardiomyopathy, and atrial fibrillation, left ventricular (LV) diastolic dysfunction plays a key role in the disease process – both as the underlying cause as well as a consequence of disease progression (Kane et al. 2011; Rosenberg and Manning 2012; Jeong and Dudley 2015). Diastolic dysfunction is characterized by an impaired relaxation and increased stiffness of the myocardium, which leads to increased filling pressures (Kovács 2015; Nagueh et al. 2016). Furthermore, several studies have noted the presence of left atrial (LA) fibrosis in patients with atrial fibrillation and other diseases that are strongly associated with diastolic dysfunction (Mahnkopf et al. 2010; Maron et al. 2014; Siontis et al. 2014). Hence, although little is known about why atrial fibrosis develops, it is likely that atrial fibrosis is at least associated with or possibly caused by elevated filling pressures.

Noninvasive diagnosis of diastolic dysfunction is a complex task that requires the comparison of a number of parameters in a comprehensive algorithm, and is clinically performed using transthoracic echocardiography (TTE) (Nagueh et al. 2016). Standard diastolic parameters in TTE include: E and A (the peak blood flow velocity through the mitral valve at the early rapid filling and atrial contraction, respectively); e′, the peak tissue velocity at the mitral annular insertion points during the early rapid filling; LA volume; tricuspid regurgitation velocity; and E/e′, which is a surrogate measure for LV filling pressure (Nagueh et al. 1998).

In cardiovascular magnetic resonance imaging (MRI), methods exist that can measure those diastolic parameters (Paelinck et al. 2005; Bollache et al. 2010; Duarte and Fernandez 2010; Leng et al. 2015; Saba et al. 2014; Seemann et al. 2017). For example, quantification of LA volumes is established (Wylie et al. 2008; Maceira et al. 2010), but there is a lack of standardized methods for MRI assessment of diastolic parameters linked to valvular flow and motion (i.e., E, A, and especially e′).

Other MRI‐based diastolic parameters have been proposed, including parameters obtained from flow patterns and tissue characterization (Ellims et al. 2012; Okayama et al. 2013; Töger et al. 2016). Atrial tissue characterization has recently become feasible noninvasively using an MRI method for visualizing fibrosis as LA late gadolinium enhancement (LGE) (McGann et al. 2008; Gatehouse et al. 2010). Little is known about why atrial fibrosis develops, but atrial fibrosis measured as LA LGE is hypothesized to result from elevated atrial pressure, and thus linked to diastolic dysfunction.

Therefore, the aim of this study was to evaluate the diastolic parameters E, A, and e′ using conventional cine and phase‐contrast images, for validation with TTE and LV filling pressure. Additionally, we aimed to correlate LA functional parameters and LA LGE to LV filling pressure and diastolic function and in a clinical population.

## Materials and Methods

### Study population

Consecutive patients at Yale New Haven Hospital who had atrial fibrosis evaluation by 3D LGE MRI for diverse indications between 2012 and 2016 were retrospectively included (53 ± 16 years, 39 females) in this IRB‐approved study. Medical charts were reviewed for TTE and invasive pressure examinations. Exclusion criteria were a nondiagnostic 3D LGE (19%); arrhythmias during imaging; examinations performed more than 1 year from the MRI; or a major cardiac event between the examinations. A total of 105 patients were screened, out of which 59 had a TTE within 1 year of MRI, and 19 had invasive pressures within 1 year. No patients had mitral valvular disease.

### Magnetic resonance imaging

Cardiac MRI was performed at 1.5T (Aera, Siemens Healthcare, Erlangen, Germany). Short‐axis cine, four‐chamber cine, and 3D LA LGE were acquired. Phase‐contrast (PC) cine images of the three‐chamber view were acquired in 14 of the patients.

Scan parameters for cines were balanced steady‐state free precession with retrospective ECG‐triggering, TR/TE/*θ *= 3 msec/1.5 msec/60°, 30 reconstructed timeframes, resolution 1.4 × 1.4 × 8 mm^3^, field of view 270 × 360 mm. Twelve views per segment yielded a true temporal resolution of 36 msec. The image acquisition is completed within one breath hold.

LA LGE was performed with ECG‐triggered and navigator‐gated fat‐saturated 3D gradient echo inversion recovery at mid‐diastole (Peters et al. 2007), 15–25 min after administration of 0.2 mmol/kg gadolinium (Gadobutrol, Bayer Healthcare, Leverkusen, Germany). Voxel size of 1.3 × 1.3 × 3.0 mm^3^ was interpolated to 0.7 × 0.7 × 1.5 mm^3^. Additional parameters were TR/TE/*θ *= 5.3 msec/2.1 msec/15°. Field of view was 360 × 360 mm. Twenty‐seven views per segment were acquired in a ky‐centric order, with a GRAPPA‐factor of 2. Typical image acquisition takes 5 min (i.e., 300 heartbeats for 50% navigator efficiency).

Phase‐contrast images were acquired using a segmented gradient echo sequence with in‐plane flow encoding (long‐axis direction). Scan parameters were TR/TE/*θ *= 5.6 msec/2.1 msec/20°, 30 reconstructed timeframes, 1.9 × 1.9 × 6 mm^3^ resolution, field of view 270 × 360 mm. Three bipolar pairs per segment yielded an actual temporal resolution of 33.6 msec. Velocity encoding ranged between 150 and 400 cm/sec. The image acquisition is completed within one breath hold.

### Transthoracic echocardiography

Experienced sonographers performed TTE using a Phillips iE33 X5 1–5 MHz (Andover, MA) or Siemens Acuson SC2000 4V1c 1.25–4.5 MHz (Malvern, PA) device. Cardiologists with level III echocardiography training performed offline interpretation using LUMEDX HealthView (Oakland, CA). E and A were obtained from a pulsed‐wave Doppler in the four‐chamber apical view, while septal and lateral e′ were acquired using tissue Doppler. Reported E, A, and e′ values were the average of measurements in 3 consecutive cardiac cycles. The time allotted for a full TTE examination at our institution is 50 min, and the offline interpretation takes 20–30 min.

### Pressure

Nineteen patients underwent invasively measured filling pressure upon clinical indication, either with pulmonary capillary wedge pressure (PCWP) or LV end‐diastolic pressure (LVEDP).

### Volume and fibrosis quantification

**Figure 1:**
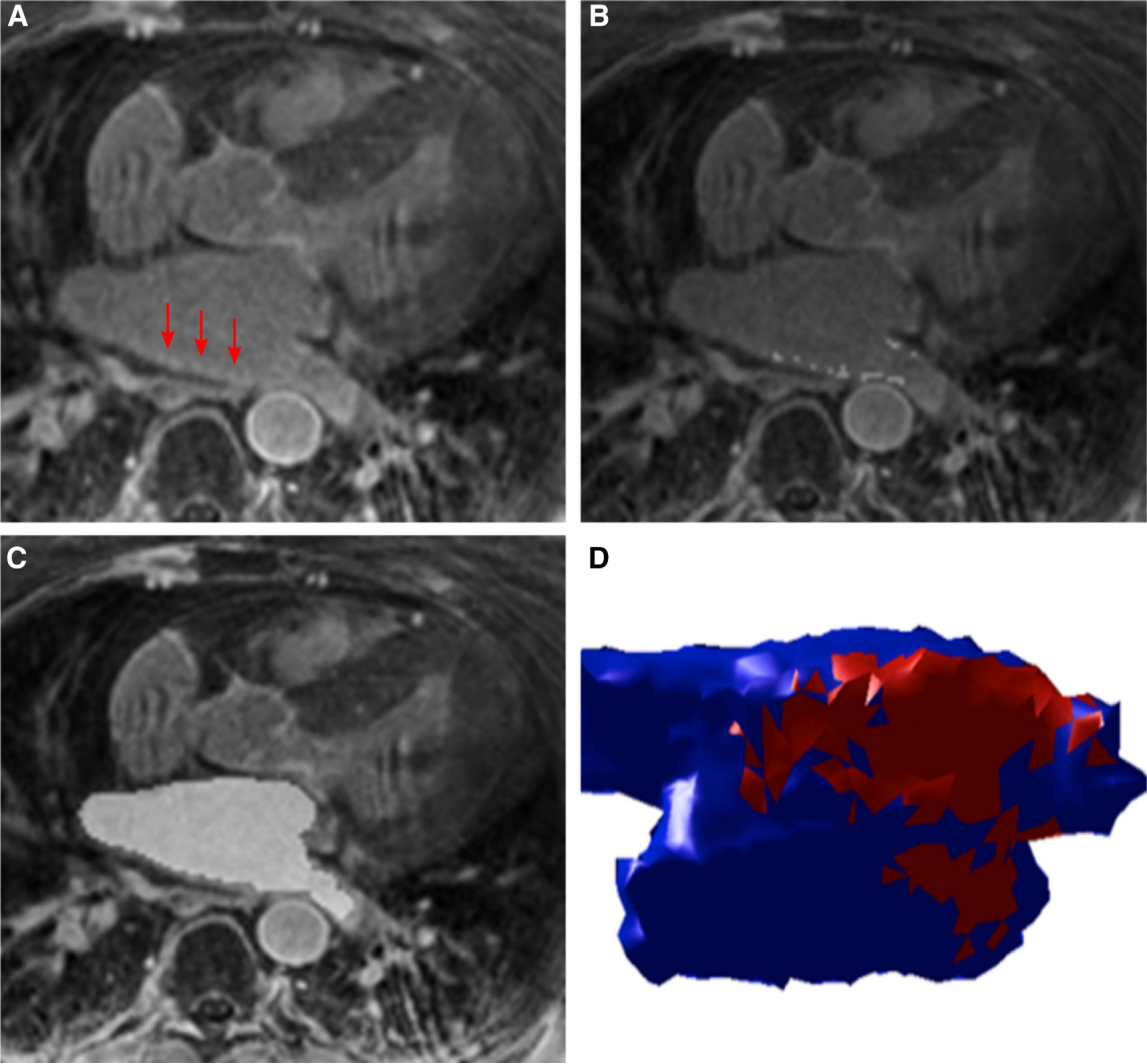
Quantification of left atrial (LA) fibrosis and volume. (A) Example of a 3D late gadolinium enhancement (LGE) image, with left atrial enhancement indicated by red arrows. (B) Segmentation of LA LGE, now displayed as light gray pixels, quantified by a semi‐automatic threshold method. (C) Segmented LA cavity in light gray, used for biplane area‐length calculation of LA volume. (D) 3D rendering of the LA, with healthy myocardium in blue and fibrosis in red.

Atrial LGE volume was quantified in a blinded fashion by D.C.P in 3D SLICER 4.3.1(slicer.org), using subject specific thresholds with manual segmentation to exclude artifacts and include atrial wall (Karim et al. 2013). The segmented LGE enhancement volume was obtained using summation of segmented areas of enhancement in each axial slice (Fig. 1). This segmentation method agreed mostly strongly with expert consensus in a recent multicenter study (Karim et al. 2013). Inter and intraobserver reliability of LA LGE segmentation has shown to be good in other similar studies (Cochet et al. 2015). Typical analysis time was 4 min.

Left atrial volume and ejection fraction were approximated by the biplane area‐length method using four‐chamber cine (Wylie et al. 2008). Atrial wall volume was estimated with a wall thickness of 2.1 mm using 3D LGE imaging (Nakamura et al. 2011). The square‐root of the percentage of LA LGE volume was transformed to a normal distribution by taking the square‐root.

LV volumes, mass and ejection fraction were measured in the short‐axis by delineation of endocardial borders in each slice and phase, and epicardial borders in end‐diastole.

### Diastolic parameters from MRI

MRI‐derived diastolic parameters e′, E, A were assessed by F.S. and R.G. using Segment v2.2 R6324(http://segment.heiberg.se) (Heiberg et al. 2010; Seemann et al. 2017).

#### Peak tissue velocity: e′

**Figure 2:**
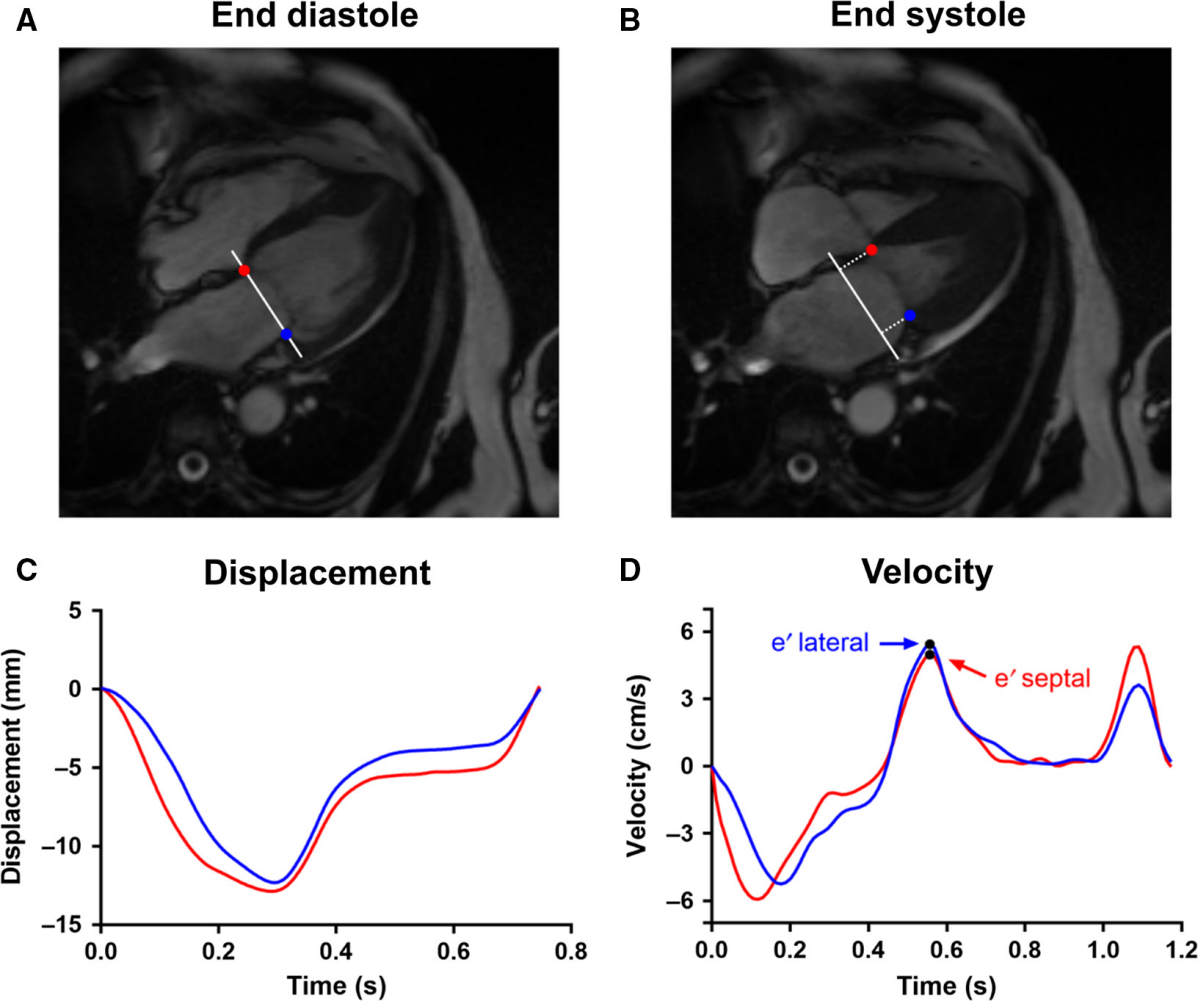
Illustration of e′ calculation. (A) The septal (red) and lateral (blue) mitral annular points were manually indicated in the end‐diastolic timeframe of the four‐chamber view. These points also define the mitral annular plane (white). Tracking of the points yields the perpendicular displacement relative to the end‐diastolic annular plane, shown in (B) for the end‐systole. (C) The resultant displacement curve for both points, septal in red and lateral in blue. (D) Velocity curve for both points, with the peak velocity (e′) indicated.

Mitral valve displacement was measured in the four chambers using a previously proposed algorithm (Seemann et al. 2017), with manual corrections when necessary, blinded to TTE data. Displacements were smoothed with splines, and velocity calculated as the time derivative. Peak velocity at early diastole yielded e′ (Fig. 2). Typical analysis time was 5 min.

#### Mitral inflow velocity: E and A

**Figure 3:**
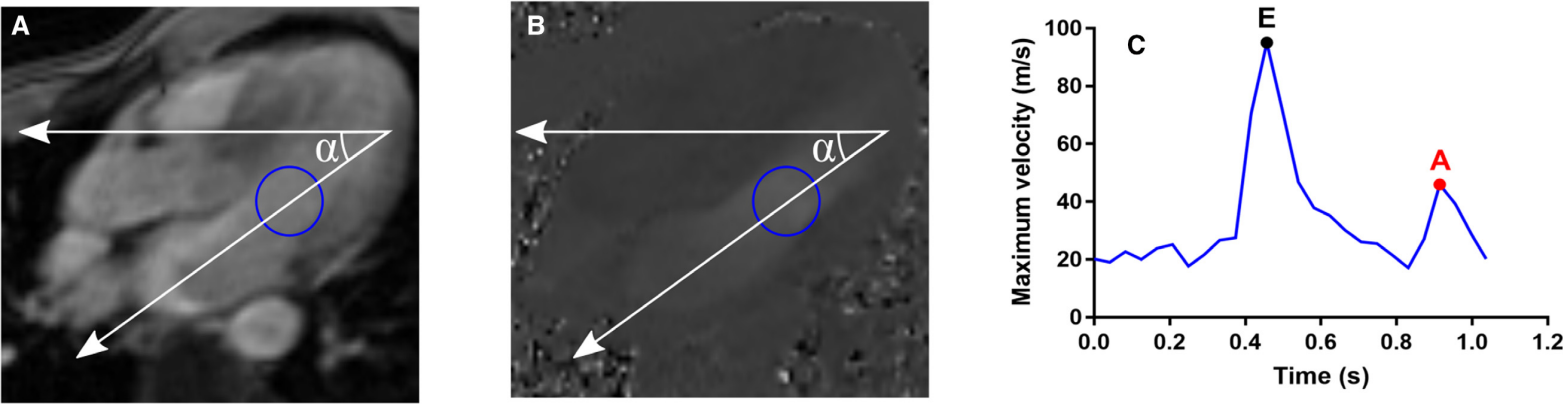
Illustration of E and A calculation. (A) Magnitude phase‐contrast image of the three‐chamber view. Blue circle indicates the region of interest in which the blood flow velocity is measured. The upper white arrow indicates the in‐plane flow encode direction, and the angled arrow passing through the region of interest indicate the flow direction through the mitral valve. Hence, the maximum velocity through the region of interest was corrected with the angle *α*. (B) Corresponding phase image with region of interest and flow directions. (C) Maximum blood flow velocity after angle correction, with E and A indicated.

E and A were quantified from PC‐MRI. A static 10 cm^2^ circular region of interest was manually placed in the mitral inflow tract. Phase offset errors were corrected based on estimation in stationary tissue (Gatehouse et al. 2010). The angle *α* between the flow encode direction and mitral inflow direction was measured. Maximum velocity was quantified as

$$v=\frac{V_{\max}}{\text{cos}\;\alpha }$$

(*V*_max_ is the maximum velocity within the region of interest). Subsequently, E and A were calculated (Fig. 3). Typical analysis time was 1 min.

#### Peak filling rate

Peak filling rate (PFR) was measured as a surrogate measure for E in patients without atrial fibrillation (*n* = 26). Volumetric flow rate was calculated as the derivative of the LV volume‐time curve. The peak flow at early diastole defined PFR. Note that E is given in m/sec, whereas PFR is given in mL/sec.

#### Validation

MRI‐derived E, A, e′, E/A, and E/e′ were validated with TTE. Average lateral and septal e′ were used for E/e′ calculation. Pressures were compared to E/e′ by MRI and TTE, and PFR/e′. PFR was compared to E by TTE.

#### Reproducibility

Interobserver and intraobserver variability in E and e′ by MRI was measured by an inexperienced observer in 10 subjects as bias ± SD and intraclass coefficient (ICC).

### Left atrial assessment

LA LGE, volume, and ejection fraction were correlated to E, A, e′, E/A, E/e′ by MRI, and pressures.

### Statistical analysis

Linear regression and Bland–Altman analysis were performed using GraphPad Prism 7.03 (GraphPad Software Inc., La Jolla, CA), with *P *<* *0.05 indicating statistical significance. Agreement between measures were considered negligible if correlation r was between 0.0–0.3, weak between 0.3–0.5, moderate between 0.5–0.7, strong between 0.7–0.9, and very strong between 0.9–1.0 (Mukaka 2012).

## Results

Subject characteristics are summarized in Table 1.

**Table 1:** Patient characteristics

	Patients with e′ by TTE and MRI *n* = 59	Patients with PC‐MRI *n* = 14	Patients with PWCP and TTE *n* = 8	Patients with LVEDP and TTE *n* = 6
Age (years)	51 ± 17	58 ± 21	38 ± 17	65 ± 14
Female (%)	37%	57%	12%	33%
BMI (kg/m^2^)	28 ± 5	28 ± 6	29 ± 8	30 ± 7
AF (%)	29%	29%	12%	17%
HCM (%)	23%	86%	12%	50%
Diabetes (%)	4%	0%	12%	0%
CAD (%)	8%	7%	12%	50%
Tx HTN (%)	54%	64%	62%	50%
LV‐EDV indexed (kg/m^2^)	85 ± 26	68 ± 21	112 ± 45	91 ± 15
LV‐EF (%)	55 ± 12	66 ± 8	41 ± 18	55 ± 9
LV mass, indexed (kg/m^2^)	69 ± 30	96 ± 32	89 ± 38	87 ± 36
LA‐vol min, indexed (mL/m^2^)	24 ± 14	20 ± 9	24 ± 13	26 ± 8
LA‐EF (%)	48 ± 17	45 ± 20	43 ± 18	43 ± 10
LA LGE (√%)	8.7 ± 10	17 ± 15	8.0 ± 5.7	9.4 ± 14

Values are given as mean ± SD. Volumes and mass were indexed to body surface area.

TTE, transthoracic echocardiography; MRI, magnetic resonance imaging; PC, phase‐contrast; PCWP, pulmonary capillary wedge pressure; LVEDP, left ventricular end‐diastolic pressure; BMI, body mass index; AF, atrial fibrillation; HCM, hypertrophic cardiomyopathy; CAD, coronary artery disease; Tx HTN, Treatment of hypertension; LV‐EDV, left ventricular end‐diastolic volume; LV‐EF, left ventricular ejection fraction; LA‐vol min, minimum left atrial volume (atrial end‐systole); LA‐EF, left atrial ejection fraction; LA LGE, left atrial late gadolinium enhancement.

### Diastolic parameter validation

#### Peak tissue velocity: e′ (*n* = 59)

**Figure 4:**
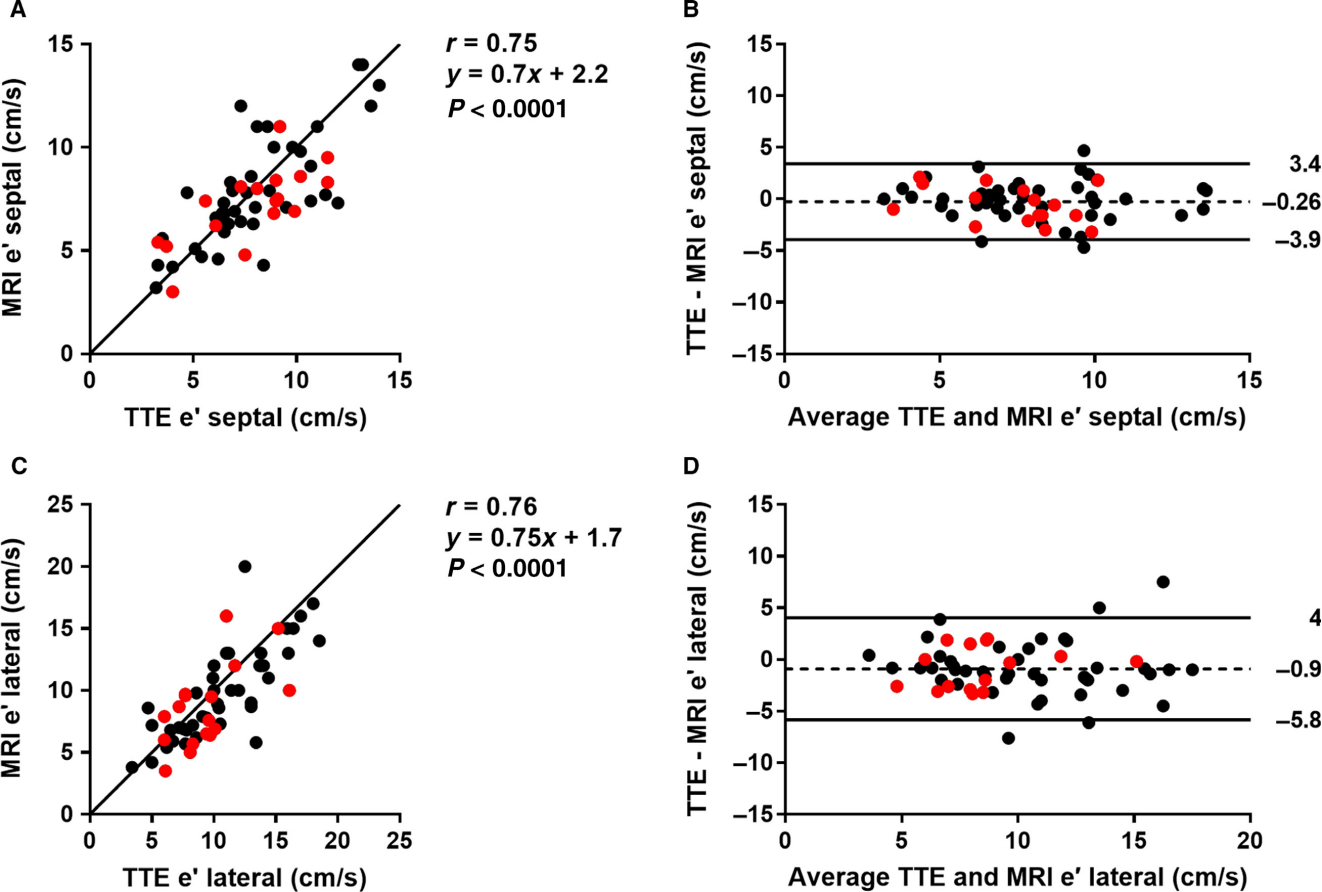
Validation of e′ by magnetic resonance imaging (MRI) with transthoracic echocardiography (TTE). (A) Scatter plot of septal e′ (*n* = 59). Red dots represent patients with atrial fibrillation. Solid line is the line of identity. (B) Bland‐Altman plot of septal e′. Solid lines represent the 95% confidence interval, dashed line the mean bias. (C) Scatter plot of lateral e (*n* = 59)′. (D) Bland‐Altman plot of lateral e′.

Validation of septal and lateral e′ by MRI with TTE is shown in Figure 4, showing a strong agreement and low bias between the image modalities (*r* = 0.75, *P* < 0.0001, bias −0.26 ±1.9 cm/sec for septal; *r* = 0.76, *P* < 0.0001, −0.9 ± 2.5 cm/sec for lateral). Furthermore, there was no linear trend when comparing the difference of TTE e′ and MRI e′ to the number of days between the TTE and MRI examinations (*r* = 0.07 for septal and *r* = −0.03 for lateral e′). This suggests that there was no systematic error added as the time between the examinations increased. Median time between MRI and TTE was 23 days, and interquartile range was 51 days.

#### Mitral inflow velocity: E and A (*n* = 14)

**Figure 5:**
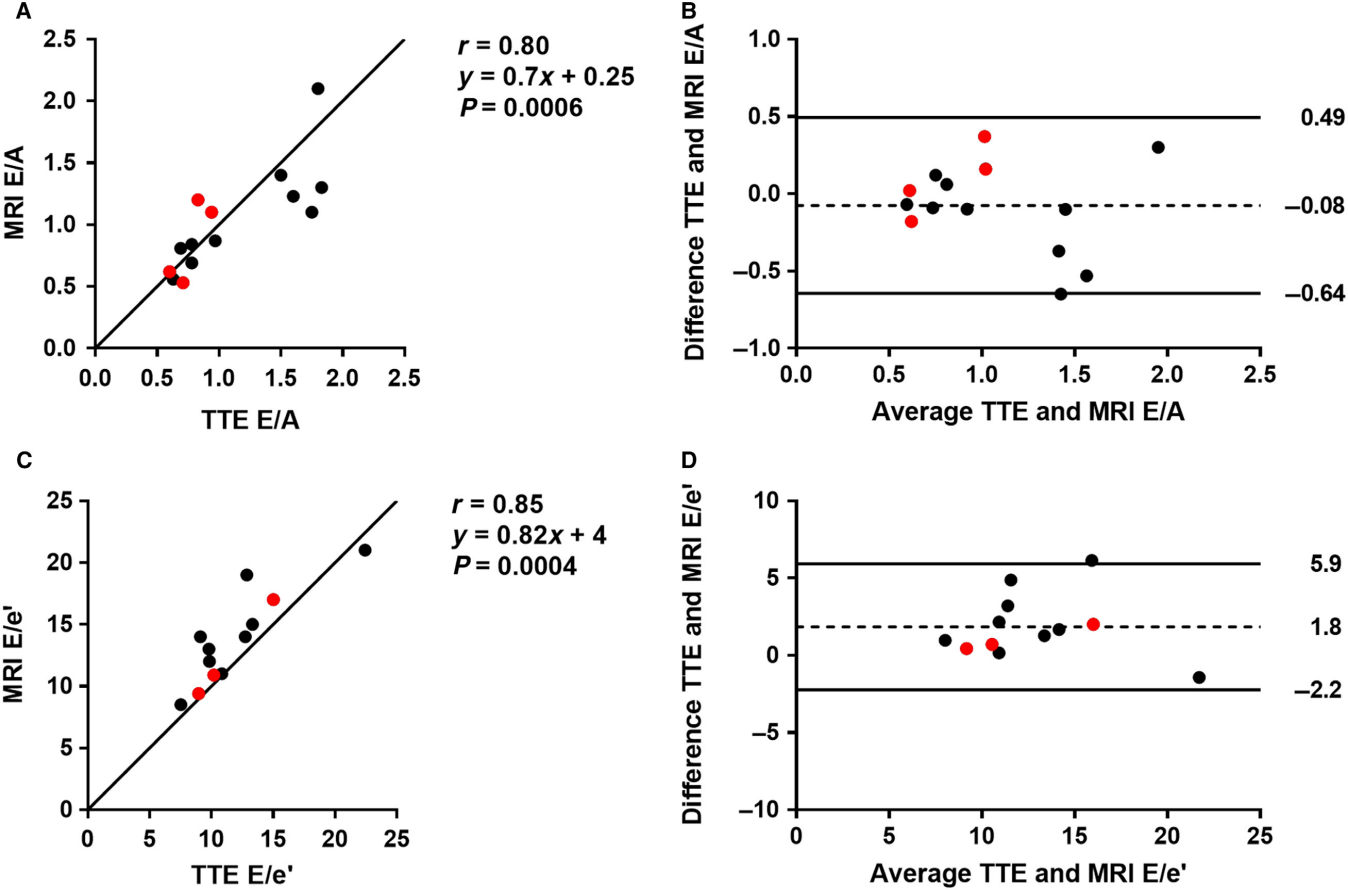
Validation of E/A and E/e’ by phase‐contrast magnetic resonance imaging (MRI) with transthoracic echocardiography (TTE). (A) Scatter plot of E/A (*n* = 14). Red dots represent patients with atrial fibrillation. Solid line is the line of identity. (B) Bland‐Altman plot of E/A. Solid lines represent the 95% confidence interval, dashed line the mean bias. (C) Scatter plot of E/e’ (*n* = 12). (D) Bland‐Altman plot of E/e’.

There was a strong agreement (*r* = 0.77, *P* = 0.001) between E by PC‐MRI and TTE, as well as for A (*r* = 0.73, *P* = 0.003). Bias and variability was low for both parameters (0.06 ± 0.11 m/sec for E and 0.10 ± 0.17 m/sec for A). The ratios E/A and E/e′ by MRI and TTE are shown in Figure 5, disclosing a strong agreement (*r* = 0.80, *P* = 0.0006 for E/A and *r* = 0.85, *P* = 0.0004 for E/e′). The average angulation *α* was 46 ± 13°. Median time between PC‐MRI and TTE was 49 days, and interquartile range was 140 days.

#### Reproducibility in MRI (*n* = 10)

Interobserver variability in septal e′ was *r* = 0.68, ICC = 0.7, bias −0.22 ± 2.0 cm/sec, and *r* = 0.70, ICC = 0.7, bias −0.33 ± 2.7 cm/sec for lateral e′. For E and A, interobserver variability was *r* = 0.88, ICC = 0.80, bias −0.08 ± 0.11 m/sec and *r* = 0.90, ICC = 0.85, bias −0.06 ± 0.12 m/sec, respectively.

Intraobserver variability was *r* = 0.83, ICC = 0.76, bias −1.07 ± 1.36 cm/sec, for septal e′, and *r* = 0.94, ICC=0.69, bias 1.5 ± 1.4 cm/sec for lateral e′. For E and A, intraobserver variability was *r* = 0.94, ICC = 0.84, bias −0.09 ± 0.08 m/sec and *r* = 0.97, ICC = 0.89, bias −0.08 ± 0.09 m/sec, respectively.

### Surrogate measure comparisons

#### Peak filling rate (*n* = 26)

The correlation between PFR and E by TTE was moderate (*r* = 0.55, *P* = 0.004). Median time between MRI and TTE was 31 days, and interquartile range was 145 days. In the cohort that had invasive pressures within a year of the MRI, the correlation between PFR and E by TTE was negligible and nonsignificant (*r* = 0.23, *P* = 0.43).

#### Pressure

The correlation between pressures (both PCWP and LVEDP) and E/e′ by TTE was moderate (*r* = 0.59, *P* = 0.03, *n* = 14). For E/e′ by PC‐MRI, the correlation was also moderate but nonsignificant (*r* = 0.52, *P* = 0.37, *n* = 5). There was no correlation between PFR/e′ (*r* = −0.006, *P* = 0.98, *n* = 19) and pressures. Median time between pressure catheterization and TTE was 89 days, and 86 days between pressure and MRI.

### Left atrial assessment

**Figure 6:**
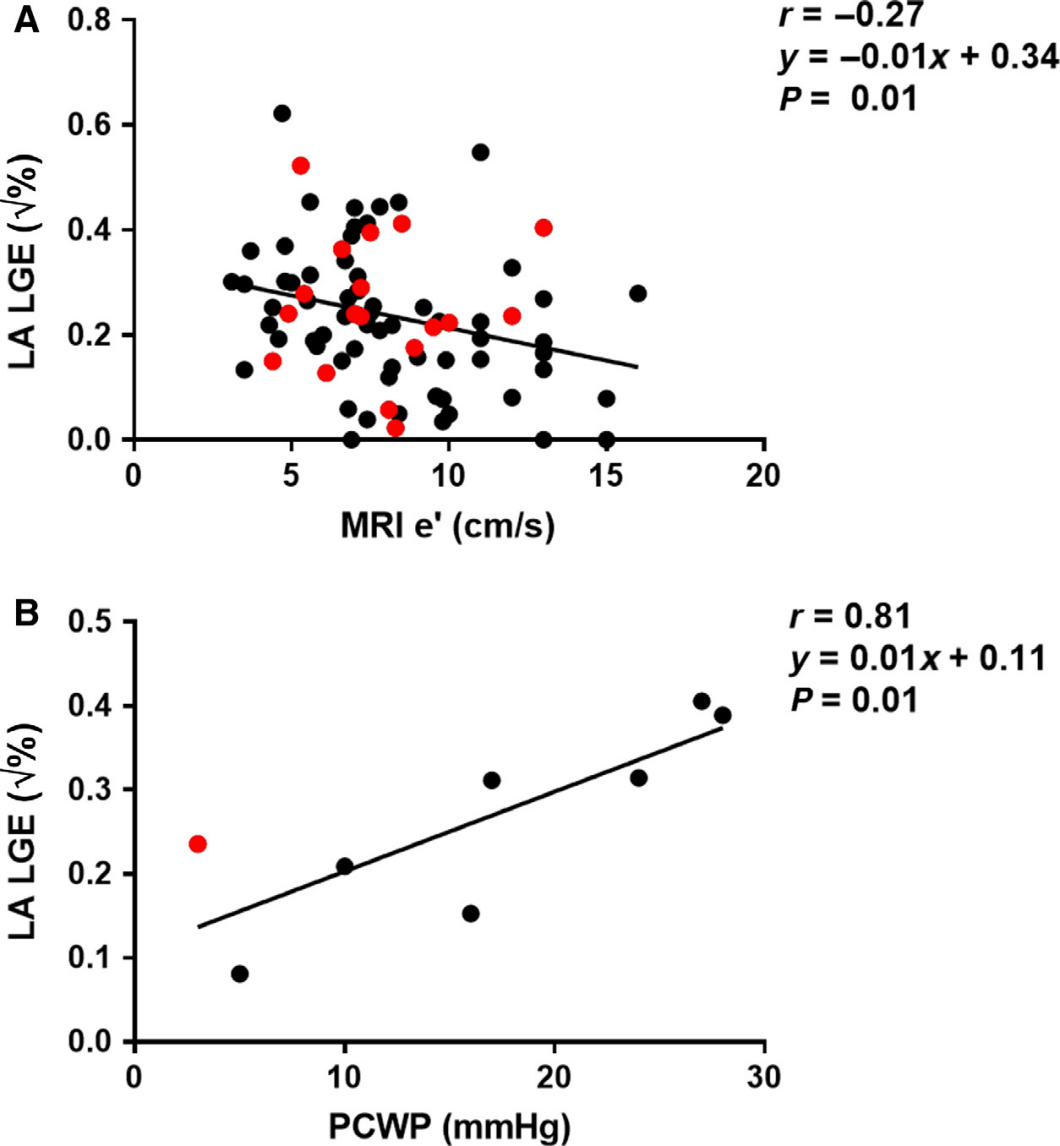
Relationship between left atrial (LA) late gadolinium enhancement (LGE) and diastolic parameters. (A) Scatter plot of square‐root of LA LGE and e’ (*n* = 86) by magnetic resonance imaging (MRI). Red dots represent patients with atrial fibrillation. Solid line is the linear regression. (B) Scatter plot of square‐root of LA LGE and pulmonary capillary wedge pressure (PCWP), *n* = 8.

The relationship between the LA assessment and diastolic parameters are summarized in Table 2, and the significant relationships to LA LGE are shown in Figure 6. There was a weak correlation between LA LGE and e′ (*r* = −0.27, *P* = 0.01), whereas the correlation between LA LGE and PCWP was strong (*r* = 0.81, *P* = 0.01). In the nonhypertrophic cardiomyopathy subgroup, correlations with LA LGE were stronger for minimum LA volume (*r* = 0.35, *P* = 0.008), filling pressure (*r* = 0.70, *P* = 0.01), and PCWP (*r* = 0.82, *P* = 0.02).

**Table 2:** Left atrial assessment

	LA LGE (√%)^a^	LA‐vol min (mL/m^2^)	LA‐EF (%)
LA‐vol min, indexed (mL /m^2^)	*r* = 0.18, *P* = 0.12		
LA‐EF (%)	*r* = −0.16, *P* = 0.15	*r* ** = −0.69, ** *P* ** < 0.0001**	
MRI e′ (cm/sec)	*r* ** = −0.27, ** *P* ** = 0.01**	*r* ** = −0.26, ** *P* ** = 0.02**	*r* ** = 0.39, ** *P* ** = 0.0003**
MRI E (m/sec)	*r* = 0.24, *P* = 0.30	*r* = 0.13, *P* = 0.58	*r* = −0.26, *P* = 0.24
MRI A (m/sec)	*r* = 0.23, *P* = 0.32	*r* = −0.19, *P* = 0.42	*r* = −0.21, *P* = 0.37
MRI E/A	*r* = −0.39, *P* = 0.08	*r* = 0.42, *P* = 0.06	*r* = −0.06, *P* = 0.78
MRI E/e′	*r* = −0.10, *P* = 0.66	*r* = 0.01, *P* = 0.95	*r* ** = −0.47, ** *P* ** = 0.03**
Filling pressure (mmHg)	*r* ** = 0.48, ** *P* ** = 0.05**	*r* = 0.16, *P* = 0.51	*r* = 0.26, *P* = 0.80
PCWP (mmHg)	*r* ** = 0.81, ** *P* ** = 0.01**	*r* = 0.54, *P* = 0.17	*r* = 0.08, *P* = 0.85
LVEDP (mmHg)	*r* = 0.26, *P* = 0.50	*r* = 0.08, *P* = 0.82	*r* = 0.24, *P* = 0.82

Filling pressure is both PCWP and LVEDP. Volumes were indexed to body surface area. LA LGE, left atrial late gadolinium enhancement; LA‐vol min, minimum left atrial volume (atrial end‐systole); LA‐EF, left atrial ejection fraction; MRI, magnetic resonance imaging; PCWP, pulmonary capillary wedge pressure; LVEDP, left ventricular end‐diastolic pressure.

Significant correlates are highlighted with a bold font.

a All patients had LA LGE to some extent.

## Discussion

It is a challenging task to understand and diagnose diastolic dysfunction. Although diastolic dysfunction is a recognized factor in several cardiovascular conditions (Duarte and Fernandez 2010; Jeong and Dudley 2015; Kovács 2015; Nagueh et al. 2016), its only well‐established assessment method is echocardiography (Nagueh et al. 2016). This study validates methods for diastolic assessment by MRI, which may provide a more complete clinical evaluation, allowing for tissue characterization and diastolic function assessment from a single examination. Furthermore, our central hypothesis, that LV filling pressure is correlated with LA fibrosis, was demonstrated using these MRI‐based measures.

Evaluation of e′ by feature‐tracking has been proposed and validated (Leng et al. 2015; Seemann et al. 2017). For example, Leng et al. (2015) obtained strong agreement when validating e′ by MRI to TTE, as did this study which used a freely available image analysis tool. Furthermore, interobserver and intraobserver variability in the proposed method for e′ quantification were in parity with reported repeatability in echocardiography (Frikha et al. 2015). Taken together, this shows that MRI‐calculated e′ is well‐correlated with TTE and reproducible.

The use of PC‐MRI tissue phase mapping with through‐plane flow encoding has also been used for e′ measurement with good agreement (Karwatowski et al. 1994; Paelinck et al. 2005; Bollache et al. 2010). The strength of phase‐contrast is that it relies less on image processing. A drawback, however, is that the image plane is static and does not measure the same tissue throughout the cardiac cycle, and a low velocity encoding also reduces the temporal resolution. Finally, these images are not currently acquired as a clinical standard. Hence, an e′ method requiring a four‐chamber cine might be better suited for clinical or retrospective studies.

In the absence of phase‐contrast, E and A can be estimated from clinically acquired MRI data (short‐axis cines), by measuring PFR. PFR has been studied as a surrogate for E (Kawaji et al. 2009; Nacif et al. 2017), but only been validated in one study where the correlation was poor (Nacif et al. 2017). Our study is the first to show a moderate correlation between PFR and E. Phase‐contrast is able to measure E and A, and through‐plane methods have been tested (Bollache et al. 2010; Duarte and Fernandez 2010). These studies noted limitations such as phase offset errors, misalignment of blood flow and the image plane, and through‐plane motion. In‐plane flow, as utilized in our study, has been the much less common approach (Karwatowski et al. 1994). In‐plane flow may provide a better estimation of E and A, since velocity can be measured in the mitral inflow tract throughout the entire heartbeat. Agreement between E, A, E/A, and E/e′ by PC‐MRI and TTE were strong in this study, and bias was low. This in combination with the low inter‐ and intraobserver variability, suggest that in‐plane flow is a robust method for E and A quantification.

The established surrogate for LV filling pressure (Nagueh et al. 1998, 2016; Andersen et al. 2017), E/e′ by TTE, had a correlation with both PCWP and LVEDP. The lack of correlation of PFR/e′ is explained by the poor correlation between E and PFR in the subgroup with pressures. Since only five subjects had both PC‐MRI and pressure, this study could not establish a significant correlation between E/e′ by MRI and pressures. The correlation with E/e′ by TTE does, however, suggest that this relationship would hold in a lager cohort, which has previously been shown (Paelinck et al. 2005).

The relationship between LA LGE and PCWP has not been shown before, and is remarkable if intuitive. This, together with the correlation with e′, suggests that LA LGE is linked to diastolic function. Although the number of patients with pressures was low and the difference between correlation and causation cannot be known, there is evidence in preclinical models that hypertension or pressure overload promotes atrial fibrosis (Kistler et al. 2006; Lau et al. 2010; De Jong et al. 2013). More studies are however needed before this found relationship between LA LGE and diastolic function can indicate if any new clinical practices could benefit patients.

The reason for a correlation between LA LGE and PCWP, but not LVEDP, might be explained by differences between these measures, which have been reported (Bitar et al. 2014). Other explanations might be that most patients with an LVEDP had hypertrophic cardiomyopathy, a cohort with a genetic propensity to develop fibrosis, or that the atrial fibrosis itself may cause a discrepancy between PCWP and LVEDP.

A study limitation is the limited number of subjects with invasive pressures, and with phase‐contrast. This did not prevent us from validating E, A, and e′, and E/e′ by MRI with TTE, but likely contributed to a lack of significance between E/e′ by MRI and pressure. A second limitation is the lack of same day examinations, which for example implies that different filling conditions could not be controlled. This is often the reality in retrospective studies with clinically acquired data. However, there was no increasing error of e′ over time. Furthermore, E and A were measured in the three‐chamber view in MRI, while the TTE was in a four‐chamber view. This might have decreased the agreement, but the strong correlation of E/A suggests that the three‐chamber view suffices. While LA LGE is commonly observed in patients with atrial fibrillation, and therefore well represented in this study, the arrhythmia in these patients compromises the assessment of diastolic function. This limitation was evaded by excluding all patients who experienced arrhythmia during the TTE or MRI examination. Furthermore, the relatively young age of the study population is a limitation since many patients undergoing diastolic assessment tend to be older.

In conclusion, MRI‐evaluation of diastolic function can be performed in four‐chamber cines and phase‐contrast images with in‐plane flow encoding. Furthermore, MRI might be able to provide a noninvasive surrogate measure for LV filling pressure. An elevated PCWP and diastolic function was also shown to be strongly related to atrial fibrosis as measured by LA LGE.

## Conflict of Interest

Einar Heiberg is the founder of the company Medviso AB, Lund, Sweden, which sells a commercial version of the Segment software. The other authors do not have any conflict of interest.
